# Vector‐Free Deep Tissue Targeting of DNA/RNA Therapeutics via Single Capacitive Discharge Conductivity‐Clamped Gene Electrotransfer

**DOI:** 10.1002/advs.202406545

**Published:** 2024-11-27

**Authors:** Jeremy L. Pinyon, Georg von Jonquieres, Stephen L. Mow, Amr Al Abed, Keng‐Yin Lai, Mathumathi Manoharan, Edward N. Crawford, Stanley H. Xue, Sarah Smith‐Moore, Lisa J. Caproni, Sarah Milsom, Matthias Klugmann, Nigel H. Lovell, Gary D. Housley

**Affiliations:** ^1^ Translational Neuroscience Facility Department of Physiology School of Biomedical Sciences Graduate School of Biomedical Engineering Tyree Institute for Health Engineering (IHealthE) UNSW Sydney NSW 2052 Australia; ^2^ Charles Perkins Centre School of Medical Sciences Faculty of Medicine and Health University of Sydney Camperdown NSW 2006 Australia; ^3^ Touchlight Genetics Ltd Lower Sunbury Road Hampton UK TW12 2ER

**Keywords:** DNA and RNA vaccines, electric field focusing, nonviral gene therapy, nucleic acid electrotransfer, precision gene delivery platform

## Abstract

Viral vector and lipid nanoparticle based gene delivery have limitations around spatiotemporal control, transgene packaging size, and vector immune reactivity, compromising translation of nucleic acid (NA) therapeutics. In the emerging field of DNA and particularly RNA‐based gene therapies, vector‐free delivery platforms are identified as a key unmet need. Here, this work addresses these challenges through gene electrotransfer (GET) of “naked” polyanionic DNA/mRNA using a single needle form‐factor which supports “electro‐lens” based compression of the local electric field, and local control of tissue conductivity, enabling single capacitive discharge minimal charge gene delivery. Proof‐of‐concept studies for “single capacitive discharge conductivity‐clamped gene electrotransfer” (SCD‐CC‐GET) deep tissue delivery of naked DNA and mRNA in the mouse hindlimb skeletal muscle achieve stable (>18 month) expression of luciferase reporter synthetic DNA, and mRNA encoding the reporter yield rapid onset (<3 h) high transient expression for several weeks. Delivery of DNAs encoding secreted alkaline phosphatase and Cal/09 influenza virus hemagglutinin antigen generate high systemic circulating recombinant protein levels and antibody titres. The findings support adoption of SCD‐CC‐GET for vaccines and immunotherapies, and extend the utility of this technology to meet the demand for efficient vector‐free, precision, deep tissue delivery of NA therapeutics.

## Graphical abstract

Vector‐free spatially controlled deep tissue gene expression is achieved using single capacitive discharge conductivity‐clamped gene electrotransfer (SCD‐CC‐GET) via an electro‐lens integrated into a needle probe. In mouse hindlimb skeletal muscle, delivery of naked reporter DNA produces stable gene expression, whereas reporter mRNA expression is faster and persists for weeks. In a proof‐of‐concept for DNA/mRNA immunotherapy applications, SCD‐CC‐GET achieves high titres of secreted protein and influenza virus epitope antibodies.

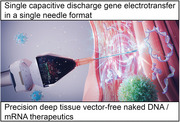

## 1 Introduction

The landscape of nucleic acid (NA) therapeutics is extending broadly across infectious disease control, treatment of noncommunicable diseases, regenerative medicine, and highly personalized treatments of gene disorders.^[^ ^1^ ^]^ The sophistication of precision gene‐based therapeutics leverages the expanding knowledge of functional genomics, AI‐based DNA/RNA design and cell‐free manufacturing technologies.^[^ ^2^ ^]^ At the center of this paradigm shift in molecular medicine are advances in NA delivery technologies matched to the therapeutic applications. The drive to develop nonviral DNA and RNA delivery platforms^[^ ^3^ ^]^ has been vindicated by the response to the COVID19 pandemic, where efficacy, development speed, and cost‐effectiveness have driven adoption. Unifying challenges of vector‐based DNA and RNA delivery approaches include their unrestricted dispersion, limited packaging capacities, and inherent immunogenicity.^[^ ^4^, ^5^ ^]^ Consequently, there is a pressing need for safe and efficacious vector‐free NA delivery platforms for modern gene medicines ranging from vaccines to personalized focal gene editing. Current methods for vector‐free in vivo gene transfer including the ballistic gene gun, sonoporation, and conventional “open field” electroporation/gene electrotransfer (GET), that mediate uptake of therapeutic NA, are typically cytotoxic, tissue damaging, and painful.^[^ ^6^ ^]^

GET is an effective gene delivery modality for “naked” NA delivery that utilizes an electric field to facilitate localized and instantaneous binding of the polyanionic NA molecules of mega Dalton size to the cathode‐facing cell membranes, where endocytosis enables DNA uptake followed by subsequent payload delivery to the nucleus for expression,^[^ ^7^ ^]^ or cytosolic protein translation with mRNA. GET is increasingly being adopted in clinical settings for applications ranging from DNA vaccines,^[^ ^8^, ^9^ ^]^ cancer treatments,^[^ ^10^, ^11^ ^]^ and gene augmentation directed at vision disorders,^[^ ^12^ ^]^ or enhancing hearing outcomes for medical bionics. The latter utilized “close‐field” GET via pulsed electric field focusing, creating an “electro‐lens” using a multielectrode bionic array for precision delivery of neurotrophin encoding NAs to the cochlea, promoting directed ectopic auditory nerve regeneration.^[^ ^13^, ^14^, ^15^, ^16^ ^]^ The efficiency of this bionic array‐directed gene electrotransfer technology (BaDGE) was enhanced several‐fold by integrating microfluidics to bias electrical conductivity to the polyanionic DNA species, described as “conductivity‐clamped gene electrotransfer” (CC‐GET).^[^ ^17^, ^18^ ^]^

Here we demonstrate a paradigm shift in precision vector‐free DNA and mRNA delivery in vivo achieved using single capacitive discharge (SCD) CC‐GET integrated into a disposable needle‐hub configuration. The development of this SCD‐CC‐GET technology proved highly efficient for stable DNA expression in skeletal muscle and achieved superior early onset protein translation with mRNA delivery. SCD‐CC‐GET is minimally invasive and enabled titratable secreted alkaline phosphatase production and immune‐priming responses against Influenza A H1N1 Cal/09 hemagglutinin encoded by synthetic DNAs. These proof‐of‐concept studies reflect the broad potential for SCD‐CC‐GET to enable precision nonviral NA therapeutics across focal gene therapy applications.

## 2 Results and Discussion

### 2.1 Optimizing ConductivityClamped Gene Electrotransfer in Skeletal Muscle

**Figure 1:**
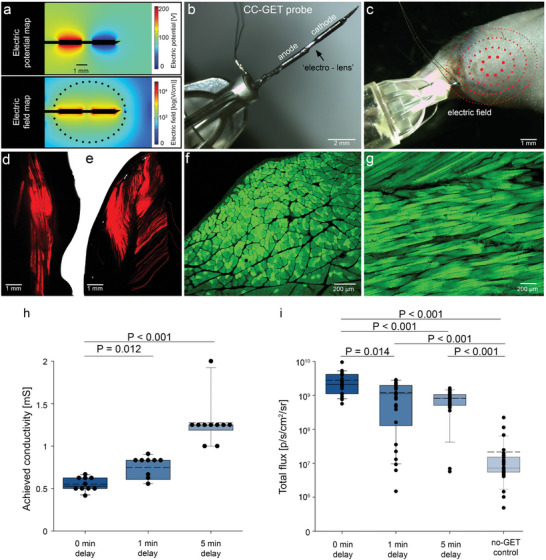
Pulsed CC‐GET of naked DNA achieves high efficiency gene expression in mouse hindlimb skeletal muscle in vivo. a) Modeled maps of electric potential and derived electric field in the mouse hindlimb muscle tissue surrounding the CC‐GET probe (COMSOL Multiphysics) based on local conductivity matched to baseline measurements, for a 200 V pulse across the electrodes. Dashed boundary in the electric field map indicates CC‐GET threshold for gene expression (≈120 V cm^−1^). b) CC‐GET probe showing “electro‐lens” established by the nonconductive space separating two Pt‐Ir electrodes (after electro‐lens patent application^[^ ^19^ ^]^). c) Image of a shaved adult mouse hindlimb with the CC‐GET probe inserted and pDNA in 10% sucrose carrier (50 µL) injected (simulated electric field overlay in red). d,e) Whole‐mount low‐power LSM images of mCherry expression in mouse hindlimb flexor digitorium longus and gastrocnemius muscle groups from two mice 7 days following CC‐GET (CMVp‐mCHERRYnls pDNA; 2 µg µL^−1^; 50 µL in 10% sucrose carrier; 1 min delay prior to delivery of 5 × 4 ms 50 mA/60 V square wave pulses). f) Transverse and g) longitudinal cryosections of the mouse gastrocnemius muscle 4 days after electrotransfer of a CAGp‐eGFP reporter plasmid (2 µg µL^−1^; 50 µL; in 10% sucrose;10 × 100 µs pulses at 500 µs intervals, 50 mA/60 V) demonstrates widespread and high efficiency gene expression in the target muscle. h) fLuc – encoding dbCMVp‐fLuc dbDNA; 2 µg µL^−1^; 50 µL in 10% sucrose was delivered into the mouse hindlimb, with electrotransfer with the CC‐GET probe 1 (5 × 4 ms, 50 mA) performed immediately (0 min), 1 min, or 5 min following injection (n = 10). A significant correlation between the achieved local conductance between the electrodes [mS] and the dbCMVp‐fLuc dbDNA incubation time prior to electrotransfer was recorded. Conductance during immediate CC‐GET (0.552 ± 0.025 mS) was significantly lower than conductance measured after 1 min (0.748 ± 0.042 mS; *p* = 0.012) and conductance increased further following a 5 min incubation of dbCMVp‐fLuc dbDNA (1.275 ± 0.087 mS; *p* < 0.001) prior to CC‐GET. This indicated that immediate pulse delivery achieves superior conductivity‐clamping and hence highest electric field strength for a fixed current pulse train. i) Luciferin–Luciferase bioluminescence imaging of dbCMVp‐fLuc dbDNA expression demonstrates that photon emission was inversely correlated to the delay between incubation time before CC‐GET pulse delivery (*n* = 30). Bioluminescence increase over the no‐GET control (2.15 × 10^7^ ± 8.05 × 10^6^) was highly significant *p* < 0.001 for each 0 min (2.79 × 10^9^ ± 3.55 × 10^8^), 1 min (1.22 × 10^9^ ± 1.80 × 10^8^), and 5 min (8.18 × 10^9^ ± 7.95 × 10^7^) incubation time before CC‐GET (bioluminescence data from 1, 3, and 7 days combined). Significantly elevated bioluminescence emission was detected using immediate post‐injection CC‐GET compared to 1 min (*p* = 0.014) or 5 min (*p* < 0.001) pulse delay after injection. Box plots show 25% and 75% percentile, median and mean (dashed lines), with 95% confidence intervals. One‐way ANOVA on ranks with Tukey post‐hoc test for all pairwise multiple comparison was performed on both data sets. Electro‐lens controlled GET described in patent application.^[^ ^19^ ^]^

The initial prototype CC‐GET “electro‐lens” probe (CC‐GET probe) was constructed as a co‐axial “needle” with anode and cathode elements separated by a nonconductive electric field focusing zone, with delivery of the NA via the tip^[^ ^13^, ^17^ ^]^ (**Figure** **1**a,b). An iso‐osmotic nonconductive carrier solution reduced local conductivity, biased to the polyanionic “naked” NA. Electric iso‐potential and electric field profiles (Figure 1a) were modeled using COMSOL for the CC‐GET probe, based on an applied voltage of 200 V via a custom‐built controller. The predicted ellipsoid‐shaped electric field profile is congruent with the in vivo gene transfer area observed in the adult C57BL/6J mouse hindlimb, where 50 µL of reporter plasmid DNA (pDNA; CMVp‐mCHERRYnls or CAGp‐eGFP pDNA; 2 µg µL^−1^) was injected, followed by delivery of 5 × 4 ms or 10 × 100 µs square wave pulses (50 mA/≈60 V) respectively; imaged at 4 and 7 days in the whole‐mount hindlimb muscle (Figure 1c–g).

Given that conductivity‐clamping efficiency was likely to depend on timing of the pulsed electric field following NA injection, this was systematically tested. CC‐GET (5 × 4 ms pulses) was performed immediately, 1 min, or 5 min after injection of a synthetic luciferase‐encoding DNA (dbCMVp‐fLuc dbDNA) into the hindlimb muscle. The control system (DS5 clinical stimulator and custom interface) captured the applied voltage required (*V*_a_) across the electrodes to drive the specified 50 mA current (where *V*_a_ is a surrogate measure of field strength, the driving force for GET), thus enabling calculation of local conductance (*G* = *V*_a_/*I*). A maximum reduction in tissue conductivity of 45% occurred immediately following injection of the DNA in the nonconductive carrier (baseline 1.01 ± 0.025 mS, *n* = 6, reduced to 0.552 ± 0.025 mS at time zero, *n* = 10; *p* < 0.001, *t*‐test). A delay of 1 min produced a significant 43% return of conductivity (0.748 ± 0.042 mS; *p* = 0.012), with baseline restored within 5 min (Figure 1h). The bioluminescence readout of expression (reporting field strength) correlated with the local conductivity analysis, with maximum expression achieved when CC‐GET immediately followed NA delivery (Figure 1i). Subsequent experiments used immediate electrotransfer. Notably, injection of 50 µL of normal saline (0.9% NaCl), a typical GET carrier, resulted in a significant increase in local conductance (1.662 ± 0.208 mS; *p* = 0.0111; *t*‐test compared to baseline), diminishing field strength for a fixed current amplitude.

### 2.2 Adoption of a Single Exponential Decay Pulse Enables Efficient “Naked” pDNA Electrotransfer

**Figure 2:**
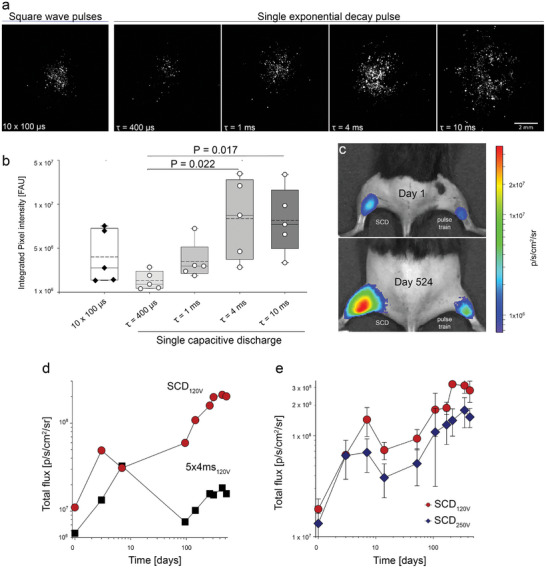
Single exponential decay CC‐GET enables field focused, titratable gene expression. a) Reference image of HEK293 cells expressing nuclear localized mCherry fluorescence 3 days following CC‐GET using a conventional 10 × 100 µs constant current (50 mA) pulse train delivered via a Digitimer DS5 stimulator (CMVp‐mCHERRYnls pDNA (2 µg µL) in 7.5% sucrose + 0.225% NaCl, pH 7.4), compared with single exponential decay CC‐GET with varying decay constants (*τ*), generated by D‐A control of an analog stimulator (DS5, Digitimer), driving the CC‐GET probe. This demonstrated titratable scaling of gene expression. The area of expression is a bioreporter for the suprathreshold electric field. b) Integrated pixel intensity in fluorescence arbitrary units (FAU) demonstrate that while the single 1 ms exponential decay pulse (3.504 ± 0.955 FAU) and the positive control using 10 × 100 µs pulses (4.009 ± 1.354 FAU) showed intermediate expression with no significant difference, the single “capacitor‐like” exponential decay with longer time constants significantly improves mCherry expression (*τ* 4 ms, 8.356 ± 2.058; *p* = 0.022 FAU; *τ* 10 ms 8.165 ± 1.674; *p* = 0.017; compared with *τ* 400 µs (1.312 ± 0.468 FAU; *n* = 5 per group; Kruskal‐Wallis one‐way analysis of variance on ranks with Tukey post‐hoc test for multiple comparisons). Box plots reflect 25% and 75% quartiles, with data overlay. Dashed lines show mean values; solid lines show the median and error bars outline the 95^th^ percentile confidence intervals. c) In vivo comparison of luciferase reporter expression using SCD‐CC‐GET versus conventional CC‐GET pulse train in the hindlimbs of a mouse with repeated bioluminescence readout out to 524 days (luciferase plasmid CAGp‐fLuc;1 µg µL^−1^ in 10% sucrose delivered via a 2.2 µF capacitor charged to 120 V (SCD‐GET controller) driving the CC‐GET probe in the left hindlimb, compared with delivery of these pDNAs through the same CC‐GET probe, via a conventional 5 × 4 ms × 120 V square wave pulse train (Digitimer DS5 stimulator) in the opposite hindlimb muscle. Following i.p. luciferin injection, bioluminescence peak was measured (IVIS Spectrum CT imaging platform). d) Time course of repeated luciferase activity bioluminescence measurements in the hindlimbs of the mouse shown in c, at 1, 3, 7, 95, 145, 201, 263, 308, and 524 days; measured as photons per second per centimeter squared per steradian (p s^−1^–cm^2^ sr^−1^). e) Repeated luciferin – luciferase bioluminescence recording on days 1, 3, 7, 14, 51, 108, 170, 215, 300, and 431 following SCD‐CC‐GET of the CAGp‐fLuc pDNA (10 µg at 0.5 µg µL^−1^) with the tethered CC‐GET probe delivering a 120 V (red) or 250 V (blue) discharge from a 2.2 µF capacitor via the SCD‐GET controller in opposite legs (mean ± SEM; *n* = 3 per group; two‐way repeated measures ANOVA; *p* = 0.253); see Table S1 (Supporting Information) for individual data.

The exceptionally low charge transfer required for efficient gene expression using CC‐GET in mouse skeletal muscle prompted evaluation of the efficacy of single exponential decay pulses. This was first tested on HEK293 cell monolayers using CMVp‐mCHERRYnls pDNA, and either a 10 × 100 µs 200 V square wave pulse train or a single 200 V exponential decay pulse with fixed time constants (*τ*) ranging from 400 µs to 10 ms (**Figure** **2**a). Quantification of the reporter fluorescence at 72 h showed the single exponential decay pulse was highly effective, with the transfected field of mCherry positive cells increasing significantly as the time constant extended from 400 µs to plateau at 10 ms (Figure 2b). The 1 ms exponential decay pulse and the 10 × 100 µs square wave pulse control showed intermediate expression.

We proceeded to examine the efficiency and longevity of SCD‐CC‐GET in vivo, in the hindlimb muscle of adult C57BL/6J wildtype mice, utilizing a custom‐built “SCD‐GET controller” for pulse delivery through the tethered CC‐GET probe to drive GET of a firefly luciferase‐encoding pDNA (CAGp–fLuc; Figure 2c–e). The CAGp‐fLuc pDNA (in nonconductive carrier solution for conductivity‐clamping) was delivered to the gastrocnemius muscle by SCD‐CC‐GET using a 120 V, 2.2 µF SCD, or using a conventional 5 × 4 ms 120 V square wave pulse train, to opposite hindlimb muscles (Figure 2c). Transgenic luciferase‐mediated conversion of intraperitoneally injected luciferin generated sustained high photon emissions, recorded from 24 h out to 524 days post‐GET (Figure 2c–e). Figure 2d graphs the total photon flux at each timepoint and demonstrates superior and extended expression following SCD‐CC‐GET compared to the 5 × 4 ms square wave pulse train. We next sought to assess the impact of the capacitor charging voltage on SCD‐CC‐GET efficiency. Both 120 and 250 V charging levels using a 2.2 µF capacitor showed comparable fLuc expression (Figure 2e), indicating tolerance across a broad range of electric field strength. The mice were behaviorally unremarkable, with normal locomotion out to 61 weeks post‐gene transfer. We note that in this study, the luciferin‐luciferase bioluminescence reporter emission intensity in the older mice was higher than the levels in the initial few weeks post‐GET. This may reflect differences in uptake of the luciferin substrate following weight‐based intraperitoneal injection (with the mice doubling weight over time), and potentially age‐dependent differences in luciferin metabolism in these older mice. Variations in K_M_, biodistribution, and pharmacokinetics of the luciferin substrate are known to affect bioluminescence output arising from luciferin oxidation catalyzed by the recombinant luciferase reporter protein.^[^ ^20^ ^]^

### 2.3 mRNA Electrotransfer to Mouse Skeletal Muscle via SCD‐CC‐GET

**Figure 3:**
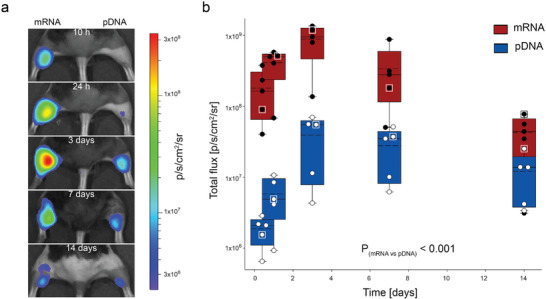
SCD‐CC‐GET drives efficient delivery of naked mRNA in skeletal muscle. a) Luciferin‐luciferase bioluminescence images from a representative mouse that received SCD‐CC‐GET (CC‐GET probe, 120 V, 2.2 µF via the SCD‐GET controller) of fLuc mRNA (left hindlimb; Trilink L‐7202 0.5 µg µL^−1^; 30 µL in 10% sucrose) and the CAGp‐fLuc pDNA (right hindlimb; 0.5 µg µL^−1^; 30 µL in 10% sucrose), 10 h, 24 h, 3 days, 7 days, and 2 weeks following GET. b) Peak photon flux over randomly paired hindlimbs from five mice receiving fLuc–pDNA (blue) and fLuc–mRNA (red) SCD‐CC‐GET (2.2 µF, 120 V). mRNA SCD‐CC‐GET resulted in a significantly stronger photon flux over the over the first 2 weeks (*p* < 0.001) due to quicker onset and substantially higher peak expression (total photon flux p s^−1^ cm^−2^ sr^−1^) 10 h mRNA: 1.81 × 10^8^ ± 0.59 × 10^7^; pDNA: 1.87 × 10^6^ ± 3.70 × 10^5^; *p* = 0.171), 24 h (mRNA: 4.14 × 10^8^ ± 9.07 × 10^7^; pDNA: 5.83 × 10^6^ ± 1.71 × 10^6^; *p* = 0.007), 72 h (mRNA: 8.82 × 108 ± 2.09 × 10^8^; pDNA: 3.93 × 10^7^ ± 1.32 × 10^7^; *p* < 0.001), and 7 days (mRNA: 3.40 × 10^8^ ± 1.42 × 10^8^; pDNA: 2.80 × 10^7^ ± 8.59 × 10^6^; *p* = 0.027). Decline of mRNA expression was evident at 14 days, from peak at 3 days, compared to relatively stable pDNA‐based expression (mRNA: 4.31 × 10^7^ ± 1.23 × 10^7^; pDNA: 1.21 × 10^7^ ± 3.99 × 10^6^; *p* = 0.802) (*n* = 5; two‐way repeated measures ANOVA with Holm–Sidak post‐hoc test for multiple comparisons; Box plots reflect 25% and 75% quartiles, with data overlay. Dashed lines show mean values; solid lines show the median and error bars outline the 95^th^ percentile confidence intervals). Data from the representative mouse shown in A are highlighted by white frames. Figure S1 (Supporting Information, top row) hematoxylin and eosin (H&E) histochemistry of target gastrocnemius muscle at the 14 day timepoint indicates an absence of pathology.

Applications for mRNA therapeutics are challenged by the constraints of nanoparticle packaging strategies to achieve plasma membrane translocation and efficient protein translation.^[^ ^4^ ^]^ Given the efficiency of pDNA delivery using the SCD‐CC‐GET platform, the efficacy of in vivo mRNA delivery to skeletal muscle was evaluated. In a randomized comparison, SCD‐CC‐GET delivered either the CAGp‐fLuc pDNA or the equivalent mRNA fLuc reporter, to opposing adult (8 weeks) mouse hindlimb muscles at equal (w/v) concentrations. Luciferase reporter expression was assayed periodically out to 2 weeks post‐GET (**Figure** **3**a,b). The study showed significantly higher mRNA over pDNA GET‐mediated fLuc expression, with rapid onset of fLuc mRNA translation and peak bioluminescence exceeding that of pDNA at the first readout time point (10 h post‐GET), reaching a 20‐fold differential over pDNA at 3 days, before decreasing.

### 2.4 Development of a Wireless SCD‐CC‐GET Gene Delivery System

**Figure 4:**
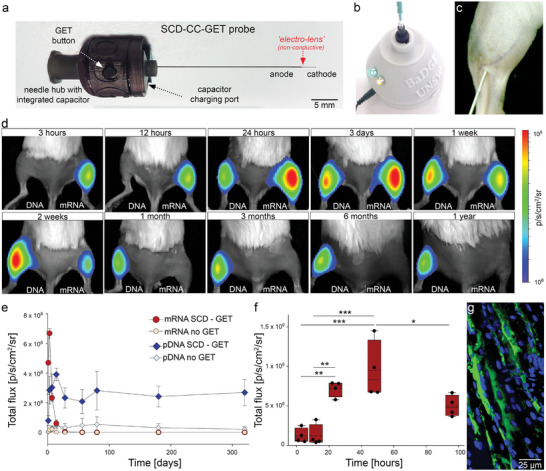
SCD‐CC‐GET needle probe/syringe device achieves rapid onset kinetics for mRNA delivery and long‐term stable expression following pDNA delivery. a) SCD‐CC‐GET probe with in‐built capacitor and coaxial insulated needle with two conductive electrodes separated by the insulated electro‐lens element and b) SCD‐GET charger, for the SCD‐CC‐GET probe (after patent application^[^ ^21^ ^]^). c) Close up of the SCD‐CC‐GET probe targeting the gastrocnemius muscle in the mouse hindlimb. d) Repeated intravital imaging of luciferin–luciferase bioluminescence at various timepoints from 3 h to ≈1 year following SCD‐CC‐GET probe‐mediated GET (2.2 µF, 200 V) of fLuc mRNA (right; 30 µL @ 0.5 µg µL^−1^ in 10% sucrose) or fLuc pDNA (left; CAGp‐fLuc, 30 µL @ 2 µg µL^−1^ in 10% sucrose) to BALB/c mouse hindlimb muscles. Images from 24 h onwards are from the same mouse. e) Time course of luciferase activity following fLuc mRNA SCD‐CC‐GET (red – 0.5 µg µL^−1^ in 10% sucrose, 2.2 µF, 200 V) fLuc pDNA SCD‐CC‐GET (blue – 2 µg µL^−1^; CAGp‐fLuc in 10% sucrose, 2.2 µF, 200 V) (*n* = 5) compared to fLuc mRNA no‐GET (cyan) and fLuc pDNA no‐GET (cream) controls (*n* = 3). Bioluminescence (total photon flux) was measured periodically at 1, 3, 7, 14, 28, 56, 80, 180, and 320 days confirming that GET is required for substantive gene expression, and that long‐term stable luciferase gene expression is achieved by pDNA SCD‐CC‐GET while mRNA SCD‐CC‐GET supports transient (weeks) albeit significantly higher expression levels (*p* < 0.001; *t*‐test) and rapid onset. mRNA‐mediated luciferase expression peaked at 6.68 × 10^8^ ± 3.19 × 10^7^ 3 days following SCD‐CC‐GET compared to pDNA driven expression reaching a peak of 3.91 × 10^8^ ± 4.17 × 10^7^ at 14 days. Data represent mean ± SEM; Table S2 (Supporting Information) for individual data. f) Onset of luciferase reporter expression following fLuc‐mRNA SCD–GET (0.5 mg mL^−1^ in 10% sucrose, 2.2 µF, 200 V). Bioluminescence (peak photon flux p s^−1^ cm^−2^ sr^−1^) was measured at 3 h (1.24 × 10^8^ ± 4.62 × 10^7^; *n* = 4), 12 h (1.24 × 10^8^ ± 6.82 × 10^7^; *n* = 6), 24 h (7.19 × 10^8^ ± 4.88 × 10^7^; *n* = 4), 48 h (9.47 × 10^8^ ± 1.83 × 10^8^; *n* = 4), and 96 h (4.99 × 10^8^ ± 6.71 × 10^7^; *n* = 4). Box plots show 25% and 75% percentile, median and mean (dashed lines), with 95% confidence intervals. ANOVA with Holm–Sidak post‐hoc test for multiple comparisons ^*^ *p *< 0.05; ^**^ *p* < 0.01, ^***^ *p *< 0.001. g) Immunofluorescence detection of recombinant luciferase (fLuc – green) in BALB/c mouse gastrocnemius muscle 24‐h after mRNA SCD‐CC‐GET (2.2 µF, 200 V). Nuclei labeled with DAPI (blue).

The minimal charge transfer required for efficient pDNA/mRNA SCD‐CC‐GET lent itself to the development of a wireless single‐use NA GET device in a minimalist form‐factor approximating a conventional needle‐syringe. This could facilitate integration of NA therapeutics into an extended range of clinical gene augmentation applications, including DNA/RNA vaccines, and intratumoral immunotherapy. We designed a SCD‐CC‐GET needle probe (SCD‐CC‐GET probe) which incorporates fluidics and SCD electronics in the needle hub (minimum dead space = 25 µL) allowing “naked” NA therapeutics delivery through attachment of a conventional low dead space syringe (**Figure** **4**a). The integration of the capacitor into the needle hub enables charging via a charging dock (SCD‐GET charger; Figure 4b). The NA is injected into the center of the tissue target via the SCD‐CC‐GET probe needle/syringe, which disperses orthogonally around the electrodes to control local tissue conductivity and maximize the electric field strength during the electric pulse (Figure 4a,c). The predetermined capacitive discharge (capacitor size and charging voltage) is triggered at the push of a button on the SCD‐CC‐GET probe hub, generating the intended electric field pulse.

We evaluated the efficiency of the SCD‐CC‐GET probe–driven gene expression in the mouse hindlimb model, comparing onset kinetics alongside longevity following delivery of pDNA (CAGp‐fLuc) versus mRNA (fLuc). This study utilized white BALB/c mice which improved the intravital bioluminescence imaging compared with the (hyperpigmented) C57BL/6J strain used in the preceding experiments. The 2.2 µF capacitor in the SCD‐CC‐GET probe hub was charged to 200 V using the SCD‐CC‐GET charger. SCD‐CC‐GET of pDNA (CAGp‐fLuc) or fLuc mRNA (equivalent number of NA molecules) was randomized across opposing hindlimb muscles. Figure 4d provides representative bioluminescence images at peak emission for each timepoint, recorded on days: 1, 3, 7, 14, 28, 56, 80, 180, and 320 (Figure 4e). mRNA SCD‐CC‐GET produced significantly higher peak bioluminescence (≈2‐fold) than pDNA, but expression was transient, falling to baseline by 28 days. Consistent with our previous study, pDNA SCD‐CC‐GET led to stable fLuc expression across the nearly 1‐year study in all animals. For both mRNA and pDNA SCD‐CC‐GET, bioluminescence in the no‐GET controls were negligible, indicative of the bio‐inert nature of the “naked” pDNA and mRNA. To resolve onset kinetics of mRNA expression, fLuc bioluminescence was assessed 3, 12, 24, 48, and 96 h post SCD‐CC‐GET using the SCD‐CC‐GET probe with an integrated 2.2 µF capacitor charged to 200 V (Figure 4f). Expression at this earliest timepoint (3 h) was substantially greater than the previously established baseline (mRNA no‐GET) at 24 h (1.24 × 10^8^ ± 4.62 × 10^7^ p s^−1^ cm^−2^ sr^−1^; *n* = 4 versus 4.94 × 10^6^ ± 2.67 × 10^6^ p s^−1^ cm^−2^ sr^−1^; *n* = 3; *t*‐test). Peak mRNA expression was reached at 48 h, declining thereafter. Recombinant fLuc protein was confirmed in the target hindlimb myocytes through immunohistochemistry 24 h post SCD‐CC‐GET (Figure 4g). H&E histology of the gastrocnemius muscles (GET target region) spanning the 3 h to 320 day timepoints was unremarkable relative to reference tissue, with the absence of macrophage (or other myeloid cell) infiltration, and comparable muscle mass and myocyte density, supporting the long‐term safety of SCD‐CC‐GET (Figure S1, Supporting Information; bottom three rows).

SCD‐CC‐GET is highly efficient at achieving translocation of pDNA and mRNA across the plasma membrane. The naked NA macromolecules (MDa) are known to require active endocytotic uptake, including clatherin‐associated endosome transport, with protein–DNA complex migration through the cytoplasm to the nucleus requiring dynein‐microtubule engagement. Nuclear pore transition in post‐mitotic cells is enhanced by interaction with nuclear localization signal containing proteins including transcription factors, which promoter and enhancer element binding sites on pDNA engage.^[^ ^7^, ^22^ ^]^ The rapid onset of luciferin‐luciferase bioluminescence reporter detection (<3 h) speaks to the efficiency of internalization and trafficking of the mRNA to the ribosomes. Onset of episomal pDNA expression took ≈12 h, and was weaker (Figures 2, 3, 4), reflecting the nuclear membrane barrier and the requirement for subsequent mRNA transcription and translation. A previous study using electro‐lens GET in the rapidly dividing HEK293 mammalian cell model estimated that on average ≈64 functional plasmid DNA copies were transported to the nucleus.^[^ ^23^ ^]^ The present in vivo studies show efficient expression in post‐mitotic skeletal myocytes, indicative of efficient nuclear pore translocation. Given that CC‐GET occurs at charge transfer levels which minimize plasma membrane electroporation,^[^ ^18^ ^]^ and in the present study, SCD‐CC‐GET is achieved in a single electric pulse of just a few ms duration, while DNA migration to the nucleus is known to take several hours, an associated increase in nuclear membrane permeability seems less likely to contribute to the efficiency of nuclear DNA delivery than the inherent effectiveness of DNA binding to the plasma membrane.

### 2.5 SCD‐CC‐GET in the Mouse Hindlimb Supports Synthetic DNA Vaccine Immunotherapy Proof‐of‐Concept

**Figure 5:**
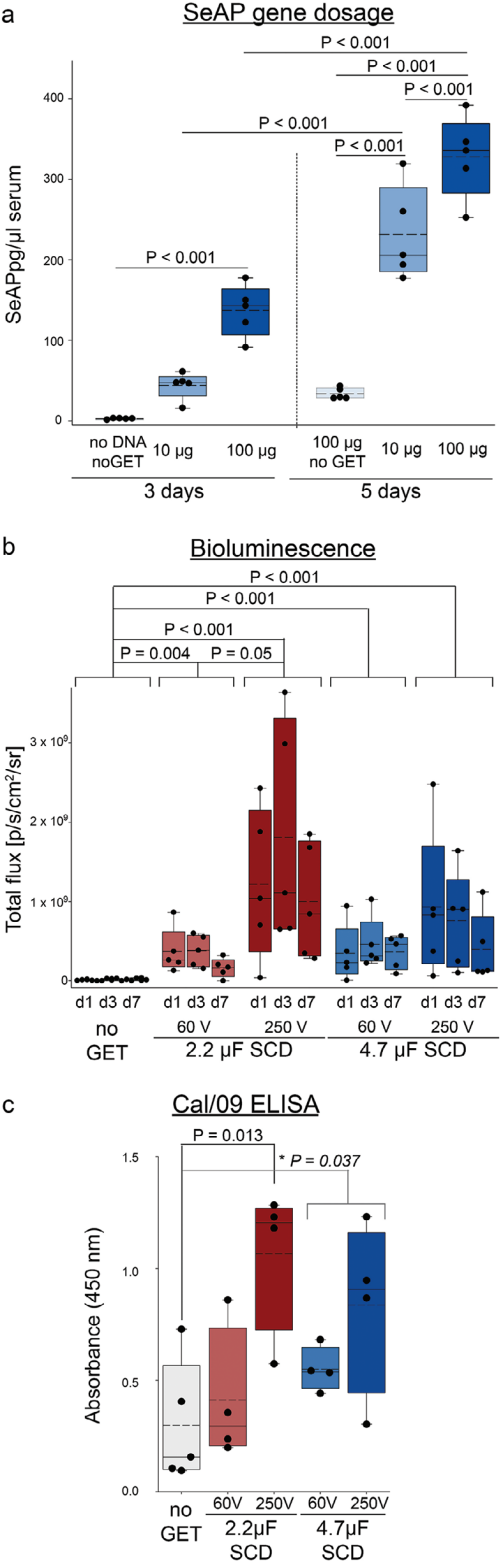
Titratable gene dose delivery via SCD‐CC‐GET for naked NA vaccines. a) SeAP serum levels measured by ELISA at 3 or 5 days (separate cohorts *n* = 5 per treatment group) following GET (2.2 µF, 120 V) of 10 µg dbCMVp‐SeAP dbDNA (20 µL; 0.5 µg µL^−1^) or 100 µg dbCMVp‐SeAP dbDNA (50 µL; 2 µg µL^−1^) in CC‐GET carrier solution. Serum SeAP levels were proportionate to the DNA concentration at 3 days (10 µg dbCMVp‐SeAP dbDNA = 44.35 ± 7.46 pg µL^−1^; 100 µg dbCMVp‐SeAP dbDNA = 136.81 ± 14.35; no DNA; no‐GET control = 2.83 ± 0.37) and increased by 5 days (10 µg dbCMVp‐SeAP dbDNA = 231.37 ± 26.00, 100 µg dbCMVp‐SeAP dbDNA = 327.91 ± 22.82; 100 µg; no‐GET control = 34.00 ± 6.90; ^***^ *p* < 0.001 two‐way ANOVA with Holm–Sidak post‐hoc test for multiple comparison). b) dbDNA luciferase bioluminescence reporter (dbCMVp‐fLuc) was delivered in a cocktail by SCD‐CC‐GET to one hindlimb, alongside dbDNA encoding an hemagglutinin antigen against the influenza Cal/09 strain (dbCMVp‐Cal/09; both DNAs 2 µg µL^−1^, 50 µL). Four GET levels were evaluated, spanning 60 V versus 250 V applied to 2.2 and 4.7 µF capacitors. Luciferase expression was measured by repeated bioluminescence imaging on day 1, 3, and 7 (*n* = 5) against a no electrotransfer control (*n* = 6). Within each individual treatment group, no significant differences in bioluminescence were detected between day 1, 3, and 7. As no significant difference was found between timepoints within experimental groups (two‐way ANOVA with Holm–Sidak post‐hoc test for multiple comparisons), data were combined. The statistically significant distinctions reflect combination of the data from the three timepoints within treatment groups (average peak photon flux p s^−1^ cm^−2^ sr^−1^: no‐GET control 1.38 × 10^7^ ± 6.09 × 10^6^; 2.2 µF – 60 V 2.24 × 10^8^ ± 1.24 × 10^8^; 2.2 µF – 250 V 1.34 × 10^9^ ± 5.81 × 10^8^; 4.7 µF – 60 V 2.89 × 10^8^ ± 1.60 × 10^8^; 4.7 µF – 250 V 6.96 × 10^8^ ± 3.77 × 10^8^). Kruskal–Wallis one‐way ANOVA on Ranks with Dunn's post‐hoc test for multiple comparisons. c) Immunosorbant assay detection of the anti‐influenza Cal/09 hemagglutinin IgG in the serum of these animals collected 7 days post SCD–GET. No electrotransfer control 0.297 ± 0.122; *n* = 5; 2.2 µF – 60 V 0.412 ± 0.153; *n* = 4; 2.2 µF – 250 V 1.067 ± 0.166; *n* = 4; 4.7 µF – 60 V 0.550 ± 0.05; *n* = 4; 4.7 µF – 250 V 0.837 ± 0.195 *n* = 4. One‐way ANOVA with Bonferroni post‐hoc test for pairwise multiple comparison. Since no statistically significant difference was detected between the 4.7 µF charging voltages, combining the data for 4.7 µF SCD‐CC‐GET at 60 and 250 V (0.693 ± 0.108) yielded a statistically significant difference to the no‐GET control (^*^Student *t*‐test with a two‐tailed *p*‐value of 0.0371). All box plots show 25% and 75% percentile, median and mean (dashed lines), with 95% confidence intervals. All experiments utilized the CC‐GET probe and SCD‐GET controller.

Current clinical trials for DNA immunotherapies utilizing clinical electroporator systems to stimulate innate immune responses show utility across oncology and DNA vaccines. Broad adoption of this NA delivery technology, particularly in the vaccine space would benefit from efficient low‐cost minimally‐invasive GET devices, inherently avoiding vector‐based side‐effects. Such GET delivery takes particular advantage from advances in rapid scalable production of synthetic DNA and mRNA. The potential for the SCD‐CC‐GET electro‐lens technology to achieve significant immunological efficacy was therefore investigated using nonplasmid‐based DNAs. Doggybone DNA (dbDNA) constructs are linear, double‐stranded, covalently closed DNA sequences^[^ ^8^, ^9^ ^]^ that have eliminated elements such as antibiotic resistance (a regulatory recommendation^[^ ^24^ ^]^). SCD‐CC‐GET was evaluated in the mouse hindlimb model for efficacy in production of secreted alkaline phosphatase (SeAP) encoded within a dbDNA construct. The gene dosing study evaluated serum levels of recombinant secreted alkaline phosphatase (SeAP) 3 or 5 days post‐SCD‐CC‐GET of 10 and 100 µg dbCMVp‐SeAP dbDNA (**Figure** **5**a). A significant threefold increase in serum SeAP levels was measured between the 10 and the 100 µg dbCMVp‐SeAP dbDNA cohorts at 3 days. In the untreated no‐dbDNA no‐SCD‐GET control, SeAP expression was negligible. In a separate cohort of animals assessed 5 days following SCD‐CC‐GET, there was a further significant increase in both the 10 µg dbCMVp‐SeAP dbDNA and 100 µg dbCMVp‐SeAP dbDNA cohorts, where the 10 µg dbCMVp‐SeAP dbDNA exceeded the day 3 dose result for 100 µg. The cohort of control dbCMVp‐SeAP dbDNA injections (100 µg) no‐GET control, exhibited minimal SeAP signal at 5 days. This experiment confirmed the capacity for SCD‐CC‐GET to drive substantial protein secretion which would facilitate systemic immunopriming for DNA vaccines.

We next sought to demonstrate that SCD‐CC‐GET could elicit an immune response and boost serum antibody levels following in vivo GET of the influenza virus Cal/09 hemaggutinin antigen coding sequence, positioning the platform for “naked” DNA and mRNA vaccine applications. The study design incorporated dual SCD‐CC‐GET delivery of dbDNA, where luciferase reporter (dbCMVp‐fLuc dbDNA) and the influenza hemaggutinin protein dbCMVp‐Cal/09 dbDNA were co‐delivered to the hindlimb in 8 to 12 week‐old C57BL/6J mice. Subsequent expression of fLuc and antibody titres generated against Cal/09‐hemagglutinin were evaluated against SCD‐CC‐GET pulse parameters. Intravital luciferin–luciferase bioluminescence was recorded on day 1, 3, and 7 following SCD‐CC‐GET and compared to a no‐GET control. All treatment groups for SCD‐CC‐GET showed significant expression compared with the no‐GET control (Figure 5b). For both 2.2 and 4.7 µF capacitors, increasing the charging voltage from 60 to 250 V produced increases in mean expression (Figure 5b). In parallel with the luciferase reporter readout in these mice, end‐point DNA vaccine antibody titres were established against SCD‐CC‐GET delivery of dbCMVp‐Cal/09 dbDNA. The serum was analyzed for Cal/09 hemagglutinin‐specific IgG content across the four GET parameters by Enzyme Linked Immunosorbent Assay (ELISA), with significantly elevated antibody titres achieved for both 2.2 and 4.7 µF – 250 V SCD‐CC‐GET groups compared to the no‐GET control (Figure 5c). The Cal/09 antibody titres were measured 7 days after SCD‐CC‐GET delivery of dbCal/09 antigen DNA. This is an early time point for evaluation of immune titres, and IgG levels can continue to rise for several weeks after antigen presentation.^[^ ^25^ ^]^ This initial proof‐of‐concept study establishes a platform for future longitudinal preclinical studies which could include booster delivery and pathogen challenges for immunoglobulin and cytokine profiling, alongside assessment of pathogen neutralization.

**Figure 6:**
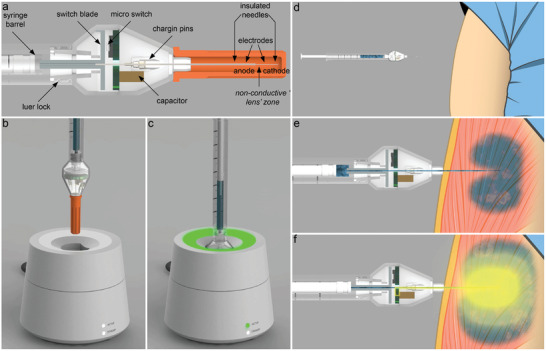
SCD‐CC‐GET electro‐lens focusing enables single pulse naked DNA/mRNA delivery in a needle/syringe‐style format. The SCD‐CC‐GET probe needle hub contains a small capacitor that on discharge enables production of a local electric field at the needle tip, driving efficient GET after local delivery of the NA therapeutic via the needle. The capacitor is charged remotely via a charging station (SCD‐GET charger), with the capacitor storing the energy until insertion of the needle into the target tissue. In a DNA/mRNA vaccine application, delivery could be intramuscular, equivalent to a conventional inoculation, where immediately following injection, the capacitor discharges to establish instantaneous GET. The “naked” therapeutic NA molecules are biologically inert unless bound to the target cells by this process, which is delineated by a critical electric field strength. a) Detail of the SCD‐CC‐GET needle hub showing the integrated capacitor, switch, charging pins and electro‐lens at the needle tip. b,c) Insertion of the SCD‐CC‐GET probe into the SCD‐GET charging station rapidly charges the device. d–f) Intramuscular SCD‐CC‐GET delivery, where the low conductivity carrier enhances the local electric field strength (conductivity‐clamping) for highly efficient gene delivery. SCD‐CC‐GET system described in patent.^[^ ^21^ ^]^

Our data establish the effectiveness of a simple disposable SCD‐CC‐GET device enabling highly efficient delivery of next generation NA vaccines and therapeutics within a predetermined tissue area defined by electro‐lens focusing of a single pulse electric field, augmented by local reduction in tissue conductivity (conductivity‐clamping) (**Figure** **6**).

### 2.6 Strategic Advantages of SCD‐CC‐GET for Deep Tissue Vector‐Free DNA/mRNA Gene Augmentation Therapeutics

NA therapeutics have broad potential to complement pharmacological treatments beyond conventional gene therapy. Central to this is efficient targeting of DNA/RNA delivery while minimizing adverse reactions. To this end, there is a strong drive to achieve vector‐free platforms.^[^ ^3^ ^]^ SCD‐CC‐GET provides significant advantages over current NA delivery modalities. Most notably are the precision spatiotemporal targeting and control of gene dosing, lack of NA packing constraints, and elimination of adverse immunological responses against the vector. Additional attributes facilitating translation include enhanced stability and reduced cost of the therapeutic, and access to conventional (non‐GMO) drug/device regulatory approval pathways.

A key point of difference for SCD‐CC‐GET within the electrotherapeutics space is the significant reduction in charge transfer required for efficient gene expression, minimizing adverse effects such as tissue injury and sustained pain. The latter is a significant adverse reaction reported by patients in clinical trials utilizing current clinical electroporators.^[^ ^26^, ^27^ ^]^ SCD‐CC‐GET has been subjectively evaluated by the team during development and found to be painless.

By way of comparison with current clinical GET devices, Figure 5b shows significant transgene expression in mouse muscle following SCD‐CC‐GET with a 2.2 µF capacitor charged to 60 V, equating a minimal 132 µC charge transfer with the sucrose‐based vehicle for local conductivity‐clamping intrinsic to the SCD‐CC‐GET gene expression efficiency.^[^ ^17^, ^21^ ^]^ Importantly, reduced local conductivity provides longer discharge time constants (*t* = RC) enhancing NA electrotransfer over the shorter time constant of “unclamped” tissue conductivity (refer to Pinyon et al., 2024,^[^ ^18^ ^]^ Figure 1j). In addition to this substantial gain in GET efficiency, the SCD‐CC‐GET needle form‐factor is likely to promote patient adoption. A review of pulse parameters used routinely in conventional “open‐field” multi‐electrode clinical electroporation devices typically reports use of several pulses of hundreds of mA for tens of milliseconds (e.g., equating to ≈78 mC charge delivery^[^ ^27^ ^]^), which is ≈600 times greater than the SCD‐CC‐GET example above. This is comparable to studies using conventional multi‐electrode GET to deliver pDNAs to mouse hindlimb muscle,^[^ ^28^, ^29^ ^]^ where despite high charge transfer requirements, the transduction efficiency was variable.

### 2.7 SCD‐CC‐GET Enables Vector‐Free DNA/mRNA‐Based Immunotherapeutics

In the DNA/RNA vaccine space, minimizing response time at the early local epidemic stage is critical for successful control of a global infectious threat to prevent pandemic spread.^[^ ^30^, ^31^ ^]^ NA vaccines are now established as core tools in the control of emerging high‐risk vector‐borne diseases.^[^ ^32^, ^33^ ^]^ Due to their selective advantages, both synthetic DNA and mRNA‐based vaccination strategies are being pursued against communicable and noncommunicable diseases with notable advances around codon optimization and synthetic vector‐free production improving antigen expression and associated antibody titres.^[^ ^34^, ^35^ ^]^ Particularly the discovery that uracil modifications can be utilized to limit inherent immunogenicity of “naked” exogenous mRNA have been pivotal for the recent expansion of mRNA vaccine research, culminating in the emergency approval of vaccines during the COVID‐19 pandemic.^[^ ^4^, ^36^, ^37^, ^38^ ^]^ In the current clinical trial landscape, mRNA vaccines have already surpassed inactivated, conjugated, attenuated, toxoid, and viral vector‐based vaccine candidates, falling second only to recombinant protein vaccines.^[^ ^39^ ^]^ Artificial intelligence driven NA design and clinical grade NA production facilities are expanding worldwide, reflecting adoption of NAs as flexible, safe, and efficacious tool for rapid local epidemic response. However, an efficient stable delivery system that overcomes the limitations of current NA packaging vectors is a pressing unmet need.^[^ ^3^, ^4^, ^40^ ^]^ While pandemic level NA vaccine responses may continue to adopt lipid nanoparticle‐based NA inoculations, rapid local response will benefit from highly efficient, rapid development and delivery, to which SCD‐CC‐GET is well matched. The current study demonstrated robust antibody titres against the Cal/09 hemagglutinin epitope encoded in the synthetic dbDNA with a single SCD‐CC‐GET treatment. It is notable that in a preceding study repeated dosing of a comparable influenza antigen encoding dbDNA (H1N1 PR8 hemagglutinin) more than doubled the immunoglobulin titres.^[^ ^8^ ^]^ Adoption of painless SCD‐CC‐GET delivery would obviate potential reluctance for booster treatments likely faced when using current clinical electroporators. Electroporation/electrotransfer has proven effective in the immunotherapy oncology space^[^ ^41^ ^]^ with currently ≈300 clinical trials listed in the USA registry.^[^ ^42^ ^]^ In clinical trial patients DNA encoding inflammatory cytokines, such as IL12, delivered to discrete melanomas by GET have been reported to achieve abscopal clearance.^[^ ^41^, ^43^ ^]^ In the broader context of vector‐free NA therapeutics, conventional multi‐needle or plate electrode configurations of clinical electroporation systems limit deep tissue applications. SCD‐CC‐GET in a scalable single needle format enables these approaches, considerably broadening the range of targets, including the potential to apply GET intratumorally, or in vulnerable tissues such as the brain, where previously pulsed electric field‐based gene delivery was prohibitive.

The long‐term reporter gene expression observed in vivo in the gastrocnemius muscle myocytes with pDNA SCD‐CC‐GET raises the question of whether this reflects (in‐part) genomic integration alongside stable extrachromosomal (episomal) expression. Genomic integration raises safety concerns across viral and nonviral DNA vaccine or other gene augmentation therapy applications, due to potential for insertional mutagenesis and tumorigenesis. Integration of vector genome varies widely across viral vectors. Although instances of adeno‐associated virus (AAV) vector‐mediated integration have been associated with oncogenesis, such as hepatocellular carcinoma in mice,^[^ ^44^ ^]^ a subset of AAV serotypes have been established as being clinically safe, despite the generally low integration rate (≈0.1%).^[^ ^5^ ^]^

A study using lipofectamine‐based transfection in HEK293 cells found that integration of supercoiled circular plasmid GFP reporter DNA occurs at significantly lower probability than linearized pDNA; based on clonal genomic sequencing after GFP expression had declined to a minima.^[^ ^45^ ^]^ While genomic integration of the circular pDNAs utilized in the present study was not specifically investigated, the high densities of transgene expressing myocytes within the bulk of the gastrocnemius muscle (Figures 1d–g and 4g), alongside maintenance of expression levels close to peak, is counter‐indicative, but warrants future consideration. The safety of electro‐lens CC‐GET delivery of circular pDNA encoding neurotrophins has been validated, with progression of the platform to a phase I/IIa first‐in‐human clinical trial for auditory nerve regeneration.^[^ ^16^, ^18^ ^]^ Long‐term expression of the dbDNA transgenes was not evaluated in the present study, however prior analysis of episome expression for generation of induced pluripotent stem cells using dbDNA supports its strong safety profile.^[^ ^46^ ^]^ Overall, the data from the current extended longitudinal studies, with no occurrence of tumorigenesis, indicate that the SCD‐CC‐GET delivery of naked DNA can achieve substantive long‐term extrachromosomal expression in nondividing cells such as myocytes and neurons with a good safety profile.

## 3 Conclusion

In conclusion, this study reports the efficiency and safety gains achieved for DNA/mRNA delivery electrotransfer using conductivity‐clamped single capacitive discharge, linked to optimization of the decay time constant of that single‐pulse electric field. The development of the SCD‐CC‐GET delivery platform for “naked” DNA and mRNA overcome significant limitations of current viral vector and liposomal modalities when precision control of gene expression is required, and is particularly well matched to the challenges in precision deep tissue targeting of NA therapeutics. In the rapidly emerging field of RNA therapeutics, achievement of vector‐free dial‐up gene dosing, where the delivery is effectively instantaneous, constrained by a highly predictable electric field volume, and nondispersing, offers significant advantages of current technologies, including re‐dosing; facilitating lower‐cost accelerated clinical translation across a wide range of gene augmentation‐based therapeutic applications.

## 4 Experimental Section

### SCD‐CC‐GET Device Manufacture

The early prototype GET device series, CC‐GET probe (Figure 1) consisted of two Pt/Ir tube electrodes (2 mm length, 400 µm outer diameter (O.D.)) bonded to an insulated 34 gauge INViSIBLE needle (TSK, Laboratory Europe, Netherlands), and connected to an external current‐regulated electric pulse controller. To manufacture these CC‐GET probes, the INViSIBLE needles (9 mm length, 200 µm O.D.,) were fully insulated with PUC polyurethane conformal coating (Electrolube, Australia; total thickness = 300 µm). Both electrodes were fixed in place with a 1 mm separation, defining the “electro‐lens”; after patent application.^[^ ^19^ ^]^ For GET a 1 mL low dead space syringe containing the DNA/mRNA was coupled to the CC‐GET probe, for local delivery of the NA solution through the tip of the needle‐type electro‐lens.

Phase 2 of the GET device prototype development (SCD‐CC‐GET probe; see Figure 4; patent application^[^ ^21^ ^]^) was based on a co‐axial stainless steel needle configuration, where inner (34 gauge with cutting bevel) and outer (28 gauge; rounded bevel) needles (Vita Needle, Company, MA, USA) were first insulated using vapor‐phase parylene C deposition (LabTop 3000 Parylene coater, Para Tech Coating). Two 2 mm length electrode surfaces, separated by the insulated 1 mm electro‐lens (as above), were then etched out using a 193 nm excimer laser, Optec PHyLS‐300, Belgium). Distal to the electro‐lens needle tip, the two concentric stainless steel needles were separately connected to a custom printed circuit board (PCB; manufactured by JLCPCB, China). This PCB, which was integrated into the GET electro‐lens needle hub, was populated with a 2.2 µF multilayer ceramic capacitor (TDK, Japan), charging port pins, and GET discharge button switch. The SCD‐GET charger for the SCD‐CC‐GET probe consisted of a custom PCB, containing a step‐up power supply from 18 V DC (external), to a controllable delivery voltage (60–300 V max; typically set to 200 V). Charging of the SCD‐CC‐GET probe, was confirmed by LED display on the SCD‐GET charger (Figure 4).^[^ ^21^ ^]^

### Modeling of the “Electro‐Lens” Electric Field Compression

Electric field potential and derived electric field maps for the muscle tissue surrounding the CC‐GET probe (Figure 1) were modeled using COMSOL Multiphysics (COMSOL AB, Sweden), where the local skeletal muscle conductivity of 0.202 S m^−1^ reflected the reported baseline conductivity measurements across the electrodes (1.01 mS). Dirichlet boundary conditions were used to define the anode and cathode. A stationary form of Maxwell's equations was solved to generate the electric potential and electric field maps. The modeled 120 V cm^−1^ electric field contour reflects the previously established threshold for GFP reporter pDNA GET established in HEK293 cell monolayers.^[^ ^18^ ^]^

### HEK293 Cell‐Based In Vitro Bioreporter Model for GET

As shown in Figure 2, HEK293 cell monolayers were used as an in vitro fluorescent bioreporter platform to quantify threshold GET electric field strengths and associated gene expression boundaries (after^[^ ^14^ ^]^), using the CC‐GET probe. Adherent HEK293 cells were grown in Dulbecco's Modified Eagle Medium (Gibco #11995‐065) supplemented with 10% fetal bovine serum, non‐essential amino acids (Thermo Fisher # 11140050), sodium pyruvate (Thermo Fisher # 11360070), and penicillin/streptamycine antibiotic (# 15140122Thermo Fisher Scientific). HEK293 cells were maintained and split with TrypLE (Thermo Fisher # 12605028) for transfection experiments by seeding onto 13 mm round glass coverslips (G400‐13, ProSciTech) 24 h prior to transfection at ≈50% cell confluence. pDNA resuspended at 2 mg mL^−1^ was delivered to the HEK293‐coated coverslips in 20 µL of 10% sucrose, with 0.5 × 10^−3^ m NaOH, and saline, with or without 50 × 10^−3^ m Tris buffer.

CC‐GET experiments used pulse trains (current‐regulated) and single exponential decay current pulses (after patent application^[^ ^21^ ^]^), generated via an isolated stimulator (DS5 Digitimer) using custom‐built waveform control and monitoring interface. Transfected coverslips were imaged for GFP reporter fluorescence by confocal microscopy 2–3 days after GET (561 nm ex., LSM710, Zeiss). Tiling was used to overlay images to capture the full expression field. Boundaries around the region of transfected cells were delineated and the area of transfection calculated using image J software (NIH, USA).

### Animals

Animal studies were conducted in accordance with the Australian Code of Practice for the Care and Use of Animals for Scientific Purposes^[^ ^47^ ^]^ and were approved by the UNSW Sydney Animal Care and Ethics Committee. C57BL/6J or BALB/c mice were housed in a temperature‐controlled room (4–5 mice per cage, separated by sex, in ventilated racks, 21–22 °C; 49–55% humidity) with 12 h light–dark cycle. Food and water were available ad libitum.

### Gene Electrotransfer to the Mouse Hind Limb Skeletal Muscles

Under isoflurane anesthesia (4% induction, 1–2% maintenance), hindlimbs of young adult C57BL/6J or female BALB/c mice (8–12‐week‐old) were shaved. A range of plasmid and fully synthetic DNAs, and mRNA reporter constructs were delivered (described in Table S3, Supporting Information) using the CC‐GET devices. For example, firefly luciferase encoding DNA (2 mg mL^−1^, dbCMVp‐Luc dbDNA, Touchlight Genetics Ltd, UK) or mRNA (0.5 mg mL^−1^, Trilink CleanCap luciferase mRNA (L‐7202)) in 10% isotonic sucrose CC‐GET carrier solution were loaded into either the CC‐GET probe or the SCD‐CC‐GET probe with a low dead space syringe, until a satellite drop appeared at the tip of the needle. The needle‐type electro‐lens was inserted into the mouse gastrocnemius muscle to the point of transition between the first and second electrode where the first electrode had completely penetrated the fascia. Then 20–50 µL of DNA or mRNA in CC‐GET carrier solution was injected into the center of the gastrocnemius muscle. The electro‐lens needle was then inserted the final 2 mm, completely penetrating the fascia of the gastrocnemius muscle, effectively placing the electro‐lens in the center of the injected NA containing CC‐GET carrier solution bolus, thereby suppressing local conductivity within the target electric field space. The subsequent electrical pulse delivery was dependent on the experimental design. Square wave pulse trains with a maximum voltage limit of 120 V set to constant current output of 50 mA were delivered by a DS5 Digitimer stimulator through a custom software developed in house. Similarly modeled SCD with fixed decay constants were driven by in‐house custom developed software. Alternatively, SCD current pulses were delivered from a 2.2 µF capacitor charged between 60 and 250 V. Note, in experiments in Figure 5, capacitors (2.2 or 4.7 µF) were coupled to the electro‐lens needle and hub via an external connector. The Trilink CleanCap luciferase base‐modified Cap‐1 mRNA (L‐7202, 1922 nucleotides) includes 5‐methoxyuridine modification and ≈120 base polyA tail.

### Luciferase Bioluminescence Intravital Imaging

For bioluminescence imaging of DNA and mRNA luciferase reporter expression, mice underwent isoflurane anesthesia (4% induction, 1–2% maintenance), hindlimbs were shaved if required and luciferin substrate was injected intraperitoneally (D‐luciferin potassium salt, Gold Biotech, USA, 150 mg kg^−1^ in normal saline) just prior to data capture (IVIS Spectrum CT, Perkin Elmer, USA). Total photon flux within a set 1 cm^2^ circular region of interest (ROI) was acquired over each hindlimb every minute (two mice at a time). Recording continued until the recorded total flux dropped for at least five consecutive measurements. The mice were removed for recovery. This was repeated periodically up to ≈18 months. Peak bioluminescence for each hindlimb was used for analysis.

### Luciferase Immunofluorescence

Immunohistochemistry was performed following standard procedures.^[^ ^48^ ^]^ Briefly, 24 h following firefly luciferase mRNA (Trilink CleanCap FLuc mRNA cat. L‐7202) SCD‐CC‐GET, the gastrocnemius muscle of the mouse was dissected and fixed in fresh 4% paraformaldehyde solution overnight. The tissue was cryopreserved in 30% sucrose and 50 µm cryosections blocked with 10% donkey serum in PBS containing 1% Triton‐X‐100 for 1 h. The tissue was then incubated with the primary anti‐fLuc antibody (Thermo Fisher Scientific # 35–6700; 1:20 dilution) in PBS containing 4% donkey serum and 0.1% Triton‐X‐100 overnight. Cryosections were then washed three times with PBS and incubated with the goat anti‐mouse IgG (H+L), Alexa Fluor 488 (Thermo Fisher Scientific #A28175; 1:1000) in PBS containing 4% donkey serum and 0.1% Triton‐X‐100 for 4 h. Sections were then counterstained with 4′,6‐diamidino‐2‐phenylindolen (DAPI), washed three times with PBS and mounted with ProLong Gold Antifade for imaging on a Zeiss Z1 AxioExaminer NLO710 confocal microscope (Carl Zeiss MicroImaging).

### Secreted Alkaline Phosphatase Assay

The chemiluminescence readout from the SeAP gene dosing study (Figure 5) was performed following the manufacturer's instructions and protocol after Best et al.^[^ ^49^ ^]^ Briefly, 8–10 week‐old C57BL/6J mice received SCD‐CC‐GET of dbCMVp‐SeAP dbDNA (10 or 100 µg; Touchlight Genetics Ltd, UK) to the left gastrocnemius muscle. At day 3 or 5 after SCD‐CC‐GET, mice were euthanized and blood was drawn by cardiac puncture and serum extracted. Colorimetric detection of the transgenic SeAP was performed using the Phospha‐Light System (Applied Biosystems; cat#T1015, USA). In a 96‐well plate, 50 µL of serum (1:50 in Dilution Buffer), or 50 µL of control SeAP enzyme, across a standard range, was applied, and 50 µL of Assay Buffer added. A total of 5 min later 50 µL reaction buffer was added and chemiluminescence read on a FlexStation 3 Multi‐Mode Microplate Reader (Molecular Devices, USA) at 0.1–1 s well^−1^. Serum SeAP concentration was calculated from the standard curve generated in Sigma Plot (Sigmaplot v. 14).

### Cal/09 Hemagglutinin Protein Assay Using ELISA

In 8–12 week‐old C57BL/6J mice under isoflurane anesthesia (as above), SCD‐CC‐GET was used for parallel delivery of luciferase reporter‐encoding synthetic DNA (dbCMVp‐Luc dbDNA, Touchlight Genetics Ltd, UK; 2 mg mL^−1^, 50 µL, SCD‐CC‐GET) into one mouse hindlimb, and synthetic DNA encoding an hemagglutinin antigen target for influenza Cal/09 (dbCMVp‐Cal/09 dbDNA, Touchlight Genetics Ltd, UK; 2 mg mL^−1^, 50 µL, SCD‐CC‐GET) into the opposite hindlimb muscle. At 7 days, following bioluminescence imaging as above, mice were euthanized, whole blood drawn by cardiac puncture, and serum extracted. Anti‐Cal/09 antibodies against the Cal/09 hemagglutinin protein were measured by direct colourimetric ELISA following Hettinga et al.^[^ ^50^ ^]^ Maxisorp 96‐well plates (DIS‐971‐010P (439454), Nunc) were coated with 50 µL of 10 µg mL^−1^ Cal/09 protein (IT‐003‐SW12p, Immune Technology Corp, New York, USA) at 4 °C overnight. Wells were then washed six times with PBS containing 0.05% Tween20 and blocked with 50 µL of Pierce Protein‐Free (PBS) Blocking Buffer (37572, ThermoFisher Scientific) for 1 h at RT. A total of 100 µL of serum (1:100 in PBS) was then added to each well and incubated for 2 h at room temperature, then washed with PBS six times and the goat anti‐mouse HRP secondary antibody (# 31430, ThermoFisher Scientific 1; 10.000) applied. Following repeated six washes, 50 µL of Ultra‐TMB HRP substrate (PN34028, ThermoFisher Scientific) was applied and the colorimetric reaction stopped after 20 min by addition of 50 µL of stop solution (0.5 m H_2_SO_4_) to each well and absorbance at 450 nm determined immediately on a, FLUOstar Omega plate reader (BMG LabTech, Australia).

### Statistics

Data were analyzed for normal distribution and where appropriate, statistical *t*‐tests (single comparisons) or ANOVA, including one or two‐way (repeated measures) ANOVA with Holm–Sidak or Bonferroni‐corrected multiple pair‐wise post‐hoc comparisons, were performed. Statistical significance was determined using alpha value = 0.05; no data were excluded as outliers (evaluated using the Grubb's test (ESD, extreme studentized deviate; PRISM)). Nonparametric statistical comparisons were undertaken when data failed the normal distribution inspection. The data were then transformed and analyzed using Kruskal–Wallis one way analysis of variance on rank, with Tukey, or Dunn's, post‐hoc tests for multiple comparisons (Sigmaplot v.14, SysStat, Germany).

## Conflict of Interest

G.D.H., J.L.P., N.H.L., E.N.C., A.A.A., and G.v.J. are inventors on cited patents assigned to UNSW Sydney. This relates of the UNSW trademark BaDGE. Patents: US Patent No. US 11,213,671 B2; US 2022/0054827 A1; US 2024/0100324 A1. S.S.‐M., L.J.C., and S.M are affiliated with Touchlight Genetics Ltd., manufacturer of the synthetic doggybone DNA constructs.

## Author Contributions

J.L.P. and G.v.J. contributed equally to this work. J.L.P. and G.v.J. contributed equally to the design and execution of the experimental work and data analysis. G.v.J., G.D.H., and J.L.P. wrote the drafts of the manuscript with input from the other authors. S.L.M., A.A.A., K‐Y.L., and M.M. undertook the primary SCD‐CC‐GET device prototype design and fabrication supported by N.H.L., G.D.H., and J.L.P. E.N.C. contributed to the design and construction of the SCD‐CC‐GET charger supported by S.L.M., G.D.H., N.H.L, and J.L.P. A.A.A. undertook the GET electro‐lens modeling of electric field profiles. S.X. contributed to the mCherry reporter expression studies in mouse hindlimb. S.S‐M., L.J.C., and S.M. from Touchlight Genetics Ltd. supported the doggybone DNA – studies in design and analysis, including luciferase, SeAP and Cal/09 studies. M.K. contributed to the design, analysis, and manuscript. Project administration by G.D.H., J.L.P., and G.v.J. Primary supervision by G.D.H.

## Supporting information



Supporting Information

## Data Availability

The data that support the findings of this study are available in the supplementary material of this article.
