# Supplementary material for: Vector‐Free Deep Tissue Targeting of DNA/RNA Therapeutics via Single Capacitive Discharge Conductivity‐Clamped Gene Electrotransfer
Source: Adv Sci (Weinh). 2024 Nov 27;12(3):2406545. doi: 10.1002/advs.202406545 (PMC11744645; doi:10.1002/advs.202406545)

ADVANCED SCIENCE
Open Access

Supporting Information

for *Adv. Sci.*, DOI 10.1002/advs.202406545

Vector-Free Deep Tissue Targeting of DNA/RNA Therapeutics via Single Capacitive Discharge Conductivity-Clamped Gene Electrotransfer

*Jeremy L. Pinyon, Georg von Jonquieres, Stephen L. Mow, Amr Al Abed, Keng-Yin Lai, Mathumathi Manoharan, Edward N. Crawford, Stanley H. Xue, Sarah Smith-Moore, Lisa J. Caproni, Sarah Milsom, Matthias Klugmann, Nigel H. Lovell and Gary D. Housley**

SUPPORTING INFORMATION

VECTOR-FREE DEEP TISSUE TARGETING OF DNA / RNA THERAPEUTICS VIA SINGLE CAPACITIVE DISCHARGE CONDUCTIVITY-CLAMPED GENE ELECTROTRANSFER

Jeremy L. Pinyon[1,2*], Georg von Jonquieres[1*], Stephen L. Mow[1], Amr Al Abed[1], Keng-Yin Lai[1], Mathumathi Manoharan[1], Edward N. Crawford[1], Stanley H. Xue[1], Sarah Smith-Moore[3], Lisa J. Caproni[3], Sarah Milsom[3], Matthias Klugmann[1], Nigel H. Lovell[1], Gary D. Housley[1¥]

[1]Translational Neuroscience Facility, Department of Physiology, School of Biomedical Sciences, Graduate School of Biomedical Engineering, Tyree Institute for Health Engineering (IHealthE), UNSW Sydney, NSW 2052, Australia
[2]Charles Perkins Centre, School of Medical Sciences, Faculty of Medicine and Health, University of Sydney, NSW 2006, Australia
[3]Touchlight Genetics Ltd, Hampton, TW12 2ER, United Kingdom

* Contributed equally to this work ¥Corresponding author: g.housley@unsw.edu.au

Supporting Information Content Summary

Table S1: 120 V vs 250 V SCD-CC-GET comparison of pDNA luciferase bioluminescence reporter expression (Data for Figure 2e).

Table S2: pDNA vs mRNA comparison of luciferase bioluminescence reporter expression (Data for Figure 4e).

Figure S1: Supporting figure showing gastrocnemius muscle histology associated with SCD-CC-GET delivery of mRNA or pDNA encoding luciferase reported in Figs. 3 and 4

Table S3: List of Plasmid and synthetic DNA and mRNA molecules

Data for Figure 2e

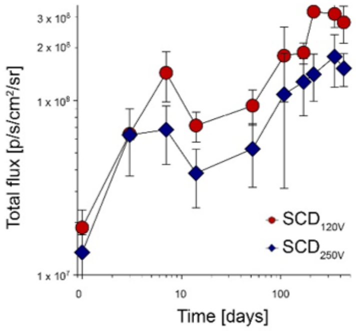

Figure 2e Legend: Repeated luciferin - luciferase bioluminescence recording on days 1, 3, 7, 14, 51, 108, 170, 215, 300, and 431 following SCD-CC-GET of the CAGp-fLuc pDNA (10 µg at 0.5 µg/µl) with the tethered CC-GET probe delivering a 120 V (red) or 250 V (blue) discharge from a 2.2 µF capacitor via the SCD-GET controller in opposite legs (mean ± SEM; n = 3 per group; two-way repeated measures ANOVA; P = 0.253).

Table S1. 120 V vs 250 V SCD-CC-GET comparison of pDNA luciferase bioluminescence reporter expression

	Total photon flux (p/cm^2/s/sr)						
**Day**	**120V - SCD-CC-GET**				**250V - SCD-CC-GET**		
1		2.01E+07	9.53E+06	2.64E+07	1.87E+07	1.49E+07	6.84E+06
3		6.69E+07	5.51E+07	7.06E+07	9.30E+07	8.68E+07	1.05E+07
7		2.26E+08	6.65E+07	1.40E+08	1.08E+08	7.41E+07	2.20E+07
14		6.73E+07	4.99E+07	9.79E+07	3.81E+07	6.29E+07	1.39E+07
52		1.15E+08	5.04E+07	1.15E+08	8.10E+07	6.56E+07	1.18E+07
108		1.59E+07	2.70E+08	2.56E+08	1.02E+07	2.61E+08	5.48E+07
170		2.37E+08	1.49E+08	1.78E+08	1.82E+08	1.66E+08	3.59E+07
215		3.45E+08	2.81E+08	3.43E+08	2.20E+08	1.32E+08	7.29E+07
349		3.24E+08	2.21E+08	3.94E+08	2.74E+08	1.92E+08	7.05E+07
431		2.76E+08	1.67E+08	3.98E+08	2.06E+08	1.60E+08	9.29E+07

Luciferin-luciferase bioluminescence measured using IVIS® Spectrum CT platform (Perkin Elmer, USA)

Data for Figure 4e

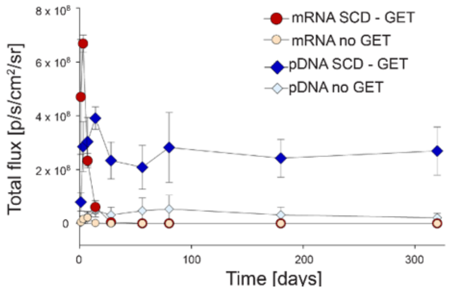

Figure 4e Legend: Time course of luciferase activity following fLuc mRNA SCD-CC-GET (red - 0.5 µg/µl in 10 % sucrose, 2.2 µF, 200 V) fLuc pDNA SCD-CC-GET (blue - 2 µg/µl; CAGp-fLuc in 10 % sucrose, 2.2 µF, 200 V) (n = 5) compared to fLuc mRNA no-GET (cyan) and fLuc pDNA no-GET (cream) controls (n = 3). Bioluminescence (total photon flux) was measured periodically at 1, 3, 7, 14, 28, 56, 80, 180 and 320 days confirming that GET is required for substantive gene expression, and that long-term stable luciferase gene expression is achieved by pDNA SCD-CC-GET while mRNA SCD-CC-GET supports transient (weeks) albeit significantly higher expression levels (P <0.001; t-test) and rapid onset. mRNA - mediated luciferase expression peaked at 6.68 x 10^8 ± 3.19 x 10^7 3 days following SCD-CC-GET compared to pDNA driven expression reaching a peak of 3.91 x 10^8 ± 4.17 x 10^7 at 14 days. Data represent mean ± SEM.

Table S2. pDNA vs mRNA comparison of luciferase bioluminescence reporter expression

	Total photon flux (p/cm^2/s/sr)							
**Day**	**mRNA + SCD-CC-GET**					**mRNA no SCD-CC-GET**		
1	3.93E+07	1.54E+07	1.16E+09	4.59E+08	6.77E+08	9.36E+06	5.34E+06	1.27E+05
3	7.24E+08	5.60E+08	7.41E+08	6.64E+08	6.53E+08	1.03E+07	3.90E+07	2.46E+05
7	2.58E+08	1.82E+08	3.27E+08	1.86E+08	2.13E+08	2.06E+05	6.50E+07	5.61E+05
14	5.07E+07	3.93E+07	1.54E+08	2.03E+07	4.04E+07	8.01E+05	3.92E+06	1.24E+05
28	3.47E+06	4.17E+06	1.14E+07	1.74E+06	2.31E+06	1.05E+05	2.88E+05	9.76E+04
56	3.84E+05	3.56E+05	3.29E+05	2.32E+05	1.16E+05	4.92E+04	9.65E+04	1.16E+05
90	6.49E+05	4.32E+05	1.33E+05	1.59E+05	6.49E+04	6.88E+04	1.18E+05	9.14E+04
180	6.43E+05	3.86E+05	3.01E+05	2.17E+05	1.36E+05	1.27E+05	7.34E+04	1.44E+05
320	5.27E+05	7.74E+05	1.79E+05	2.20E+05	1.08E+05	4.96E+04	2.81E+04	6.82E+04
**Day**	**pDNA + SCD-CC-GET**					**pDNA no SCD-CC-GET**		
1	2.85E+06	1.54E+07	1.01E+08	1.80E+08	1.12E+08	1.21E+07	1.22E+05	2.50E+05
3	1.72E+08	5.60E+08	1.32E+08	6.46E+08	2.80E+08	3.04E+07	1.69E+05	1.69E+06
7	2.26E+08	1.86E+08	1.94E+08	6.62E+08	2.52E+08	1.34E+07	2.52E+06	6.34E+07
14	3.08E+08	3.67E+08	4.60E+08	3.06E+08	5.15E+08	7.15E+07	6.34E+04	7.28E+07
28	4.74E+08	2.91E+08	1.74E+08	1.45E+08	8.59E+07	6.48E+06	1.09E+05	8.86E+07
56	5.30E+08	1.38E+08	1.15E+08	1.70E+08	9.19E+07	8.44E+05	1.02E+05	1.42E+08
90	7.99E+08	1.80E+08	1.08E+08	1.84E+08	1.41E+08	5.18E+06	1.64E+05	1.56E+08
180	5.18E+08	2.02E+08	2.06E+08	1.59E+08	1.28E+08	6.65E+06	1.00E+05	8.91E+07
320	5.21E+08	4.43E+08	1.84E+08	9.92E+07	9.91E+07	1.16E+07	5.27E+04	5.28E+07

Luciferin-luciferase bioluminescence measured using IVIS® Spectrum CT platform (Perkin Elmer, USA)

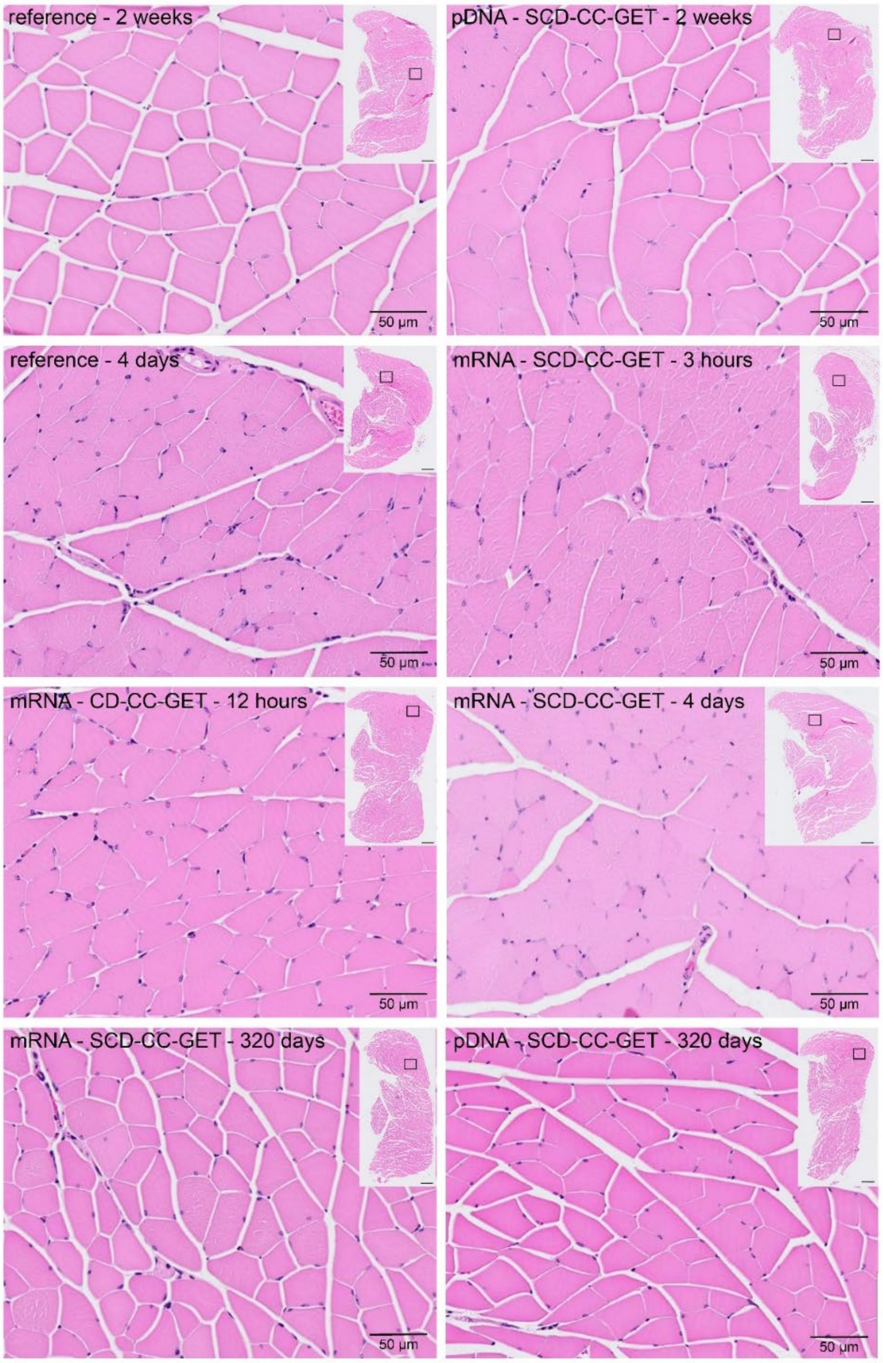

Figure S1. Gastrocnemius muscle histology associated with SCD-CC-GET delivery of mRNA or pDNA encoding luciferase reported in Figures 3 & 4. *Haematoxylin and Eosin (H&E) histochemistry on mouse hindlimb muscle treated with SCD-CC-GET delivery of pDNA or mRNA encoding luciferase across time, as shown in Figs. 3 & 4. All muscle samples were found to be histologically unremarkable with comparable muscle mass, myocyte density, and lack of macrophage infiltration indicating an absence of a broad innate immune response; supporting general safety of SCD-CC-GET. Top left reference is an untreated muscle from a C57BL/6J mouse two weeks after GET to the opposite leg. Upper right is a matching muscle that received the CAGp-fLuc pDNA, with expression as shown for 2 weeks survival in Figure 3. Lower panels are histology samples from mice in the second comparative temporal comparison of mRNA vs. DNA expression undertaken in BALB/c mice (Figure 4). The reference – 4 days, is taken from the same mouse as the mRNA - 4 days SCD-CC-GET muscle. The bottom two panels are from the same mouse, where one hindlimb received the mRNA and the opposite hindlimb received pDNA; as shown in Figure 4, where the luciferase expression was maintained in the pDNA-treated leg out to 320 days, whereas expression had ceased in the mRNA - treated leg by one month - post SCD-CC-GET delivery. The hindlimbs were fixed in paraformaldehyde, ~ 1 cm of the entire hindlimb muscle was dissected, including the gastrocnemius muscle which was the target for GET (the largest hindlimb muscle). The tissue sample included soleus. The tissue samples were paraffin embedded, sectioned (4 µm thin sections), underwent histochemical processing for H&E staining, followed by clearing and mounting. The slides were imaged using an Aperio XT slide scanner. No subsequent image processing was performed. Scale bars for low power images = 0.5 mm. Insets within the low-power images indicate the region of interest in the gastrocnemius muscle shown at high magnification.*

Table S3. List of Plasmid and synthetic DNA and mRNA molecules

CMVp-mCHERRYnls pDNA	mCherry fluorescent reporter; plasmid id: UNSW-TNF CMVp-mCHERRYnls; 4485 bp
CAGp-eGFP pDNA	Enhanced green fluorescent protein reporter; plasmid id: UNSW-TNF pMK70-CAGp-eGFP; 7088 bp
CAGp–fLuc pDNA	Luciferase reporter for luciferin-luciferase bioluminescence; plasmid id: UNSW-TNF pMK-CAG-fLuc; 6604 bp
dbCMVp-fLuc dbDNA™	Luciferase reporter for luciferin-luciferase bioluminescence; synthetic DNA id: proTLx Lux firefly luciferase, Touchlight Genetics Ltd, UK; 2555 bp
dbCMVp-SeAP dbDNA™	Secreted alkaline phosphatase; synthetic DNA id: proTLx SeAP, Touchlight Genetics Ltd, UK; 3610 bp
dbCMVp-Cal/09 dbDNA™	Cal/09 influenza virus haemagglutinin antigen; synthetic DNA id: proTLx Cal/09, Touchlight Genetics Ltd, UK; 2603 bp
mRNA fLuc	Luciferase reporter mRNA for luciferin-luciferase bioluminescence; id: Trilink CleanCap® luciferase mRNA (L-7202); 1922 nucleotides

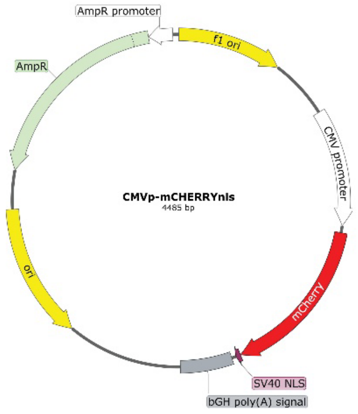

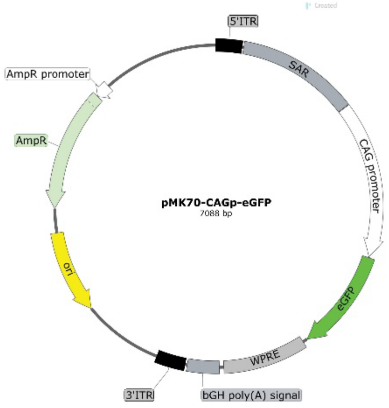

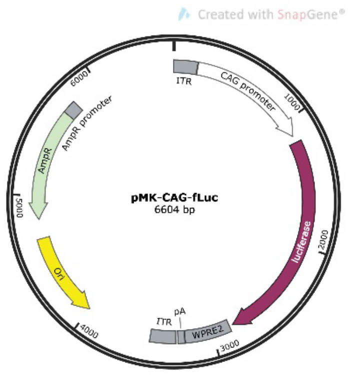

Supplement: Supplementary file 1 — Supporting Information [file ADVS-12-2406545-s001.pdf]
